# High-Resolution EEG Source Localization in Segmentation-Free Head Models Based on Finite-Difference Method and Matching Pursuit Algorithm

**DOI:** 10.3389/fnins.2021.695668

**Published:** 2021-06-28

**Authors:** Takayoshi Moridera, Essam A. Rashed, Shogo Mizutani, Akimasa Hirata

**Affiliations:** ^1^Department of Electrical and Mechanical Engineering, Nagoya Institute of Technology, Nagoya, Japan; ^2^Department of Mathematics, Faculty of Science, Suez Canal University, Ismailia, Egypt; ^3^Center of Biomedical Physics and Information Technology, Nagoya Institute of Technology, Nagoya, Japan

**Keywords:** electroencephalogram, sparse reconstruction, volume conductor model, finite difference method, inverse problem, tissue segmentation

## Abstract

Electroencephalogram (EEG) is a method to monitor electrophysiological activity on the scalp, which represents the macroscopic activity of the brain. However, it is challenging to identify EEG source regions inside the brain based on data measured by a scalp-attached network of electrodes. The accuracy of EEG source localization significantly depends on the type of head modeling and inverse problem solver. In this study, we adopted different models with a resolution of 0.5 mm to account for thin tissues/fluids, such as the cerebrospinal fluid (CSF) and dura. In particular, a spatially dependent conductivity (segmentation-free) model created using deep learning was developed and used for more realist representation of electrical conductivity. We then adopted a multi-grid-based finite-difference method (FDM) for forward problem analysis and a sparse-based algorithm to solve the inverse problem. This enabled us to perform efficient source localization using high-resolution model with a reasonable computational cost. Results indicated that the abrupt spatial change in conductivity, inherent in conventional segmentation-based head models, may trigger source localization error accumulation. The accurate modeling of the CSF, whose conductivity is the highest in the head, was an important factor affecting localization accuracy. Moreover, computational experiments with different noise levels and electrode setups demonstrate the robustness of the proposed method with segmentation-free head model.

## Introduction

An electroencephalogram (EEG) records electrical signals originating from brain activities. When the brain is activated, a minor current perpendicular to the cortical surface is generated (Mazziotta et al., 2001). An EEG measures the electric potentials on the scalp via a set of attached electrodes, for example, 21 electrodes in the international 10–20 system, and 75 electrodes in the international 10–10 system (Jurcak et al., 2007). Because each area of the brain has a unique function, it is important to identify the position of the activated area corresponding to practical actions and/or activities. This technique has various applications in diagnosis, psychology, neuroscience, brain–computer interfaces, and haptics. Among others, substantial studies have been reported on the diagnosis for epilepsy (Placantonakis and Schwartz, 2009; Tsougos et al., 2019; Razavi et al., 2020). The synaptic source activity in the cortical layer is conventionally modeled based on an electric dipole source, where the physical position can be estimated by solving an inverse problem (Nunez and Silberstein, 2000; Nunez et al., 2019).

In neuroscience analysis, as well as in several medical applications, it is crucial to estimate the EEG source location with high accuracy. Therefore, it is necessary to consider head models with more detailed anatomical structures [e.g., cerebrospinal fluid (CSF), blood vessels, etc.] for high-resolution localization. One of the limitations of employing such high-resolution models is the computational burden of solving forward problems via conventional approaches. Finite-difference or finite element methods are conventionally implemented to solve forward problems (Saleheen and Ng, 1997; Vanrumste et al., 2001; Zhang et al., 2004; Cho et al., 2015; Cuartas Morales et al., 2019; Miinalainen et al., 2019). However, the studies performed in this regard in the last decade have mainly adopted low-resolution models because of limited computational resources.

Owing to the current progress in computational electromagnetics, the successive over-relaxation (SOR) method (Hadjidimos, 2000), with the geometric multi-grid method as a preconditioner, was successfully applied to solve finite-difference problems, such as those entailed in brain stimulation applications (Laakso and Hirata, 2012b). Several computational methods have also been proposed to reduce computation time (Makarov et al., 2018).

Numerous techniques are used to localize brain wave sources via an inverse problem (Lantz et al., 1999; Zhukov et al., 2000; Whittingstall et al., 2003). A commonly used localization technique entails the lead field matrix (LFM) (Wang et al., 1992; Weinstein et al., 2000; Fuchs et al., 2002; Song et al., 2015; Nielsen et al., 2018). The LFM is a projection matrix that demonstrates the ratio between the current density (electric field) in the brain and the potentials at the electrodes attached to the scalp. A large number of equations corresponding to the test dipoles are required to construct the LFM. The current density in the brain is then estimated using a linear inverse filter or LFM-based reconstruction algorithms.

Alternative localization techniques include the minimum norm (MN) method (Wang et al., 1992; Dale and Sereno, 1993; Matsuura and Okabe, 1995), low-resolution brain electromagnetic tomography (LORETA) (Pascual-Marqui et al., 1994, 2002; Fuchs et al., 2002), and standardized LORETA (sLORETA) (Pascual-Marqui, 2002). A limitation of the MN method is that it is unsuitable for deep-source localization, as it tends to select a solution with a source on the surface (Grech et al., 2008; Costa et al., 2015). Accordingly, LORETA and sLORETA have been developed to address such limitations. However, these methods require computational memory proportional to the square of the number of head model voxels [O(*N*^2^)]. In the case of high-resolution EEG localization, this requirement limits feasible implementation. In addition, techniques based on linear inverse filter exhibit blurred distributions of computed current density (Pascual-Marqui, 2002).

As the brain activity is known to be localized and with sparse nature, sparse signal processing to EEG localization has been demonstrated (Liu et al., 2004; Xu et al., 2007; Yong et al., 2008). To date, several sparse reconstruction methods have been proposed. Among them, the matching pursuit (MP) algorithm (Mallat and Zhang, 1993) is a simple sparse algorithm that requires computational time and memory storage solely proportional to the number of voxels [O(*N*)]. In addition, this algorithm is effective for high-resolution computations.

In this study, we focus on a sparse-based EEG localization algorithm for detailed source estimation in a high-resolution anatomical model. For high-resolution localization in realistic head models, we propose an EEG source localization method that adopts the SOR and multi-grid finite-difference methods (FDM) in the forward problem analysis and the MP algorithm to solve the source localization problem, respectively. The localization performance of the proposed method is demonstrated for the head model with a spatial resolution of 0.5 mm × 0.5 mm × 0.5 mm, with approximately 27 million voxels. In addition, variations in localization performance based on variations in human head models are discussed to investigate the factors that affect localization accuracy.

## Materials and Methods

### Human Head Models

**FIGURE 1:**
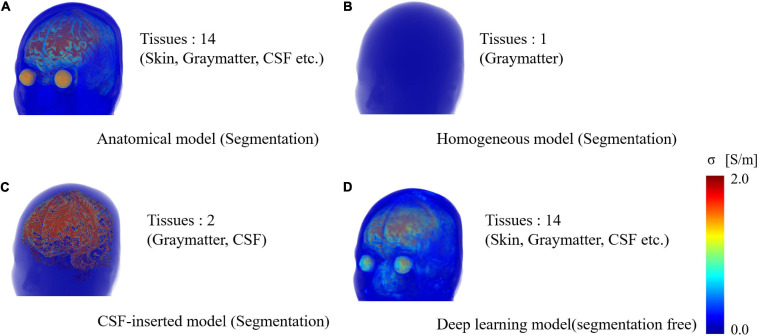
Volume rendering of numerical head models generated for single subject: **(A)** anatomical segmentation of MRI for 14 different tissues, **(B)** homogeneous model, **(C)** homogenous model with CSF-insert, and **(D)** deep-learning-estimated model (segmentation free). The resolution is 0.5 mm × 0.5 mm × 0.5 mm, and the number of voxels is 27,197,601 for all models.

**FIGURE 2:**
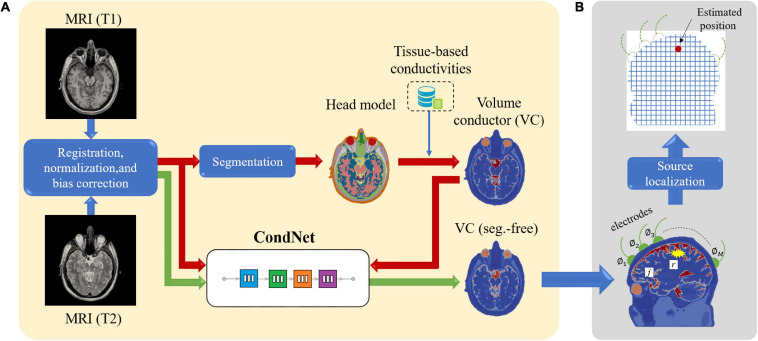
**(A)** Procedure for constructing segmentation-free head model using deep learning network (CondNet). **(B)** System model for EEG source localization.

Four models imitating a standard human male head were adopted to elucidate the effect of different factors on the localization error (Figure 1). Three segmented head models with different numbers of tissues were considered. First, an anatomical realistically shaped model was constructed from magnetic resonance (MR) images of an adult male; here, MR imaging was performed using the procedure detailed in our previous report (Laakso et al., 2015). The medical images have been acquired at National Institute of Physiological Science, Japan. The ethical approval has been obtained at Nagoya Institute of Technology (2019-014). The model was segmented into 14 tissues: skin, muscle, fat, bone (cortical and cancellous), cartilage, gray matter, white matter, cerebellum, CSF, humor, blood, mucous membrane, and dura. In the case of the second model, the realistic model was considered to consist of a homogeneous tissue with the conductivity value of gray matter. Note that the assignment of different conductivity in the single tissue does not result in any change of computed current density (electric field) distribution. In the third model, the CSF in the homogeneous realistically shaped model is considered based on the assumption that the CSF significantly influences the localization accuracy (Zanto et al., 2011). For the fourth model, a deep learning network, named CondNet as shown in Figure 2A, was adopted to automatically generate a volume conductor without segmentation, as detailed in our previous work (Rashed et al., 2020). CondNet input is MRI (T1/T2-weighted) scans with 1-mm resolution and generate the corresponding volume conductor. A feature of the model generated using CondNet is the transition of its tissue conductivity values, which is smoother than that in models generated via segmentation methods. In other words, a major advantage of this model is the suppression of impractical alterations in conductivity around tissue boundaries, which is unique among segmented head models (Reilly and Hirata, 2016). The resolution of all the models is 0.5 mm × 0.5 mm × 0.5 mm, such that the thin and detailed CSF layer is considered. Table 1 shows the conductivity of each tissue, which was determined based on the 4-Cole–Cole model presented in a previous report (Gabriel et al., 1996). The conductivity of the skin was set at 0.1 S/m, and because the value in the report is that for the stratum corneum, 0.1 S/m was suggested for practical applications.

**TABLE 1:** Conductivity of each tissue.

Tissue	Conductivity (S/m)
Skin	0.1000
Muscle	0.2020
Fat	0.0377
Bone (cort.)	0.0200
Bone (canc.)	0.0756
Cartilage	0.1611
Gray matter	0.0275
White matter	0.0277
Cerebellum	0.0475
CSF	2.0000
Humor	1.5000
Blood	0.6999
Mucous membrane	0.0004
Dura	0.5003

### Forward Problem Based on Finite-Difference Method

The scalar-potential finite-difference (SPFD) method (Dawson and Stuchly, 1996) was adopted to determine the scalar potential in this study. The scalar potential is provided by Poisson’s equation:

(1) $$\begin{array}{c} \nabla \cdot \left(\sigma \nabla \phi \right)=-\nabla \cdot J, \end{array}$$

where σ, φ, and *J* represent conductivity, scalar potential, and current density, respectively. Potentials were defined on each voxel as unknowns. By discretizing Eq. (1) with a quasi-static approximation, the potential at one node is expressed by Kirchhoff’s current law:

(2) $$\begin{array}{c} \sum_{n=1}^{6}S_{n}\phi_{n}-\sum_{n=1}^{6}S_{n}\phi_{0}=j\omega q, \end{array}$$

where *n*, *S*_*n*_, φ*_*n*_*, ω, and *q* denote the index of nodes, edge conductance from the *n*-th node to the 0-node (derived from the tissue conductivity of the surrounding voxels), potential at the *n*-th node, angular frequency of the wave source, and electrical charge at the *n*-th node, respectively. The potentials are solved by the simultaneous computation of Eq. (2) for all the nodes. Then, the current density is derived as:

(3) $$\begin{array}{c} \text{j}=\sigma \text{E}=-\sigma \nabla \phi , \end{array}$$

where ***E*** denotes the electric field. In the SPFD method, SOR (Hadjidimos, 2000) was used for fast convergence of iterative process of a linear system of equations, which is a variant of the Gauss–Seidel method. As a preconditioner, geometric multi-grid methods were adopted for the calculation. In geometric multi-grid method, coarse grids are constructed from the given fine grid (original head model with 0.5-mm resolution), and coarse grid corrections are computed using discrete systems constructed on the coarse grids (four-layer, 1, 2, 4, 8 mm) (Stüben and Trottenberg, 1982; Yavneh, 2006; Laakso and Hirata, 2012b). The number of multi-grid layers was six, and the convergence condition was defined as a relative residual smaller than 10^–6^.

### Calculation of LFM

Lead field matrix is defined as a projection matrix from current sources in the discrete gray matter to potentials measured on electrodes located on the scalp (Weinstein et al., 2000; Fuchs et al., 2002; Song et al., 2015). The elements of LFM represent the ratio of current density and potential, and are expressed as:

(4) $$\begin{array}{c} \text{Lj}=\phi , \end{array}$$

where *L* is a matrix of *M* × 3*N*, and *M* and *N* are the number of electrodes and number of gray matter voxels, respectively. In addition, ***j*** is a 3*N* × 1 vector of current density strength in each voxel and in the *x*, *y*, and *z* directions, while φ is the *M* × 1 potential vector measured by electrodes. Generally, the LFM is generated by calculating the electrical potential at the electrodes when a dipole is positioned on all voxels corresponding to gray matter. However, owing to computational cost, it is impractical to calculate the LFM for all voxels. In our head models with a resolution of 0.5 mm, 3.2 million voxels exist, which corresponding to 3 × 3.2 million cases. Therefore, the LFM is generated using the reciprocity principle (Weinstein et al., 2000; Hallez et al., 2007). First, one electrode is selected as the ground electrode. After selecting one of the electrodes other than the ground electrode, a current source is introduced to compute the electric field induced in the gray matter via the SPFD method. This procedure is repeated for the remaining electrodes to construct the LFM as follows:

(5) $$\begin{array}{c} \text{L}=\frac{1}{I}\left[\begin{array}{ccccccc} E_{1}^{1,x} & E_{1}^{1,y} & E_{1}^{1,z} & \cdots & E_{1}^{N,x} & E_{1}^{N,y} & E_{1}^{N,z}\\ E_{2}^{1,x} & E_{2}^{1,y} & E_{2}^{1,z} & \cdots & E_{2}^{N,x} & E_{2}^{N,y} & E_{2}^{N,z}\\ \vdots & \vdots & \vdots & \ddots & \vdots & \vdots & \vdots \\ E_{M}^{1,x} & E_{M}^{1,y} & E_{M}^{1,z} & \cdots & E_{M}^{N,x} & E_{M}^{N,y} & E_{M}^{N,z} \end{array}\right], \end{array}$$

where *I* is the injection current applied to two electrodes when deriving the LFM. For the international 10–10 and 10–20 systems whose electrode number is 75 and 21, respectively, the number of forward problem (electromagnetic computation) needed for generating LFM was 74 and 21.

### Inverse Problem

Figure 2B shows a schematic illustration of the system model of the EEG source localization procedure in this study. A set of *M* electrodes was positioned on the head surface, and the observed scalp potential vector was expressed as ϕ=[ϕ_1_ϕ_2_⋯ϕ_M_], where φ*_*M*_* is the potential of the *M*-th electrode. The current density distribution ***j*** is defined in the *N* gray matter voxels. We define the maximum-power location of ***j*** as ***r*** because the power of ***j*** is assumed to be concentrated in one location, as mentioned in Section “Calculation of LFM.”

The pseudocode of the algorithm used to compute ***j*** is presented in Table 2. The basic principle of this algorithm is constructing a sparse vector by iteratively selecting the most plausible basic vector from the dictionary matrix. With this approach, a sparse current density ***j*** can be calculated from ***L***, which corresponds to the dictionary matrix. Practically, the reconstructed ***j*** does not follow Kirchhoff’s current law. In this study, we assume that only one wave source is estimated in the brain, then the iteration of the algorithm is conducted once, and the maximum power location of ***j*** is obtained as ***r***.

**TABLE 2:** Pseudo code of proposed algorithm based on matching pursuit.

Set **L** as in Eq. (5)
for *i* = 1:3*N*
$corrcoef\left(i\right)=\frac{\phi \cdot L_{i}}{||\phi ||||L_{i}||}$
end for
Estimated source location: *i*^*est*^ = argmax_*i*_*corrcoef*(*i*)
Convert cell index to location *i*^*est*^→*x*^*est*^,*y*^*est*^,*z*^*est*^
(.) expresses the inner product

### Evaluation Procedure and Metrics

To evaluate the relationship between the number of electrodes and EEG source localization accuracy, the international 10–20 and 10–10 systems were considered (Jurcak et al., 2007). Assuming a cerebral activity, a small electric dipole with a length of 0.5 mm was placed in gray matter at a randomly selected position *r*_*t*_ and either one of three directions (*x*, *y*, and *z*). The electric potentials on the scalp and current density in the gray matter were calculated based on a forward problem using the SPFD method. Gaussian white noise was added to the potentials on the scalp, which affect the signal-to-noise ratio (SNR). The estimated source location was derived from the MP algorithm described in Section “Inverse Problem.” The Euclidean distance between the selected test and estimated source locations was used to define the estimation error. This process was conducted for 500 patterns to reduce the uncertainty of EEG source localization owing to Gaussian white noise. The average estimation error and its standard deviation were calculated as metrics for the localization accuracy in the four head models, as presented in Figure 1. To validate the how localization error is different when the head model is generated using different methods, two-sample *t*-test was used. The *t*-test is computed using data obtained for different SNR, and significance threshold is set to *p* < 0.05.

## Results

### Effect of Head Modelling in Forward Problem Model

**FIGURE 3:**
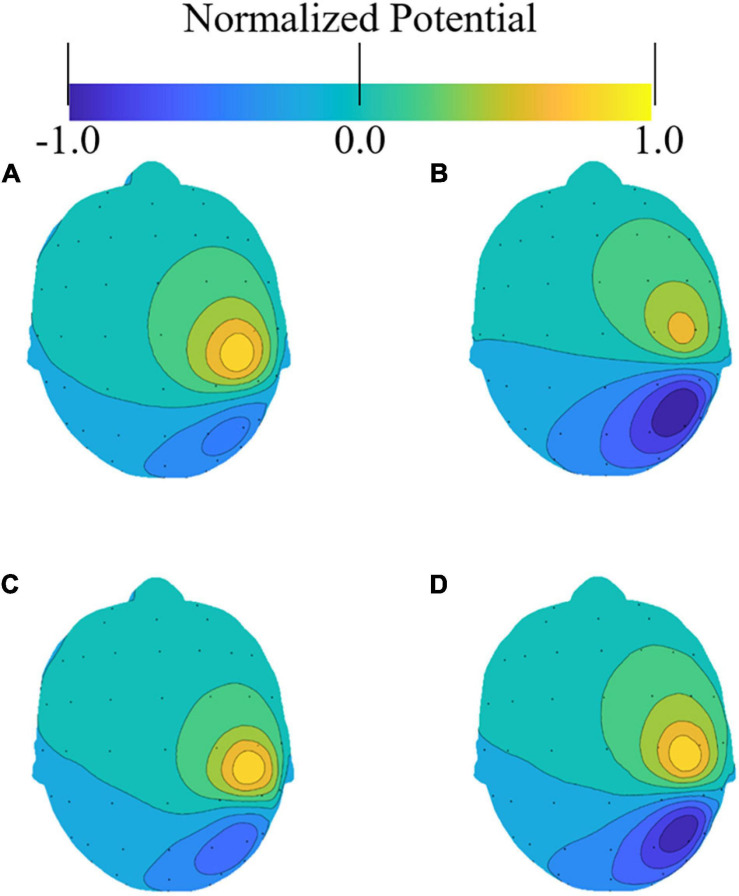
Examples of electric potential distribution on a scalp. Small dots represent the electrode locations as per the international 10–10 system. Potential distributions were normalized with the maximum value on the scalp in the **(A)** anatomical model, **(B)** its homogenized model, **(C)** CSF-insert model, and **(D)** segmentation-free model.

The effect of different head models on the accuracy of the solution to the forward problem was evaluated. Figure 3 presents an example of the induced potential distribution on the scalp of the four head models. As illustrated in the figure, all models exhibited similar distributions, although some differences were observed owing to the different conductivity distributions of the models. For the potentials on the electrodes, the correlation coefficients between the segmentation-free model and the anatomical, homogeneous, and CSF-insert models were 0.894, 0.964, and 0.913, respectively (Figure 3).

The averaged correlation coefficients between the scalp potentials induced by the 500 selected test sources were 0.902, 0.953, and 0.920 for the anatomical model, its homogeneous model, and the CSF-insert model, respectively (Table 3). The computation time of the forward problem is 30.6 s per source location with an Intel Xeon Gold-6130 at 2.20 GHz running CentOS 7.5 (16 cores, 32 threads, 96 GB RAM).

**TABLE 3:** Averaged correlation coefficient of potentials on the electrodes between each model for 500 selected test sources.

	Anatomical	Homogeneous	CSF-insert	Segmentation-free
Anatomical	1			
Homogeneous	0.850	1		
CSF-insert	0.896	0.907	1	
Segmentation-free	0.902	0.953	0.920	1

### Evaluation of Estimation Performance

**FIGURE 4:**
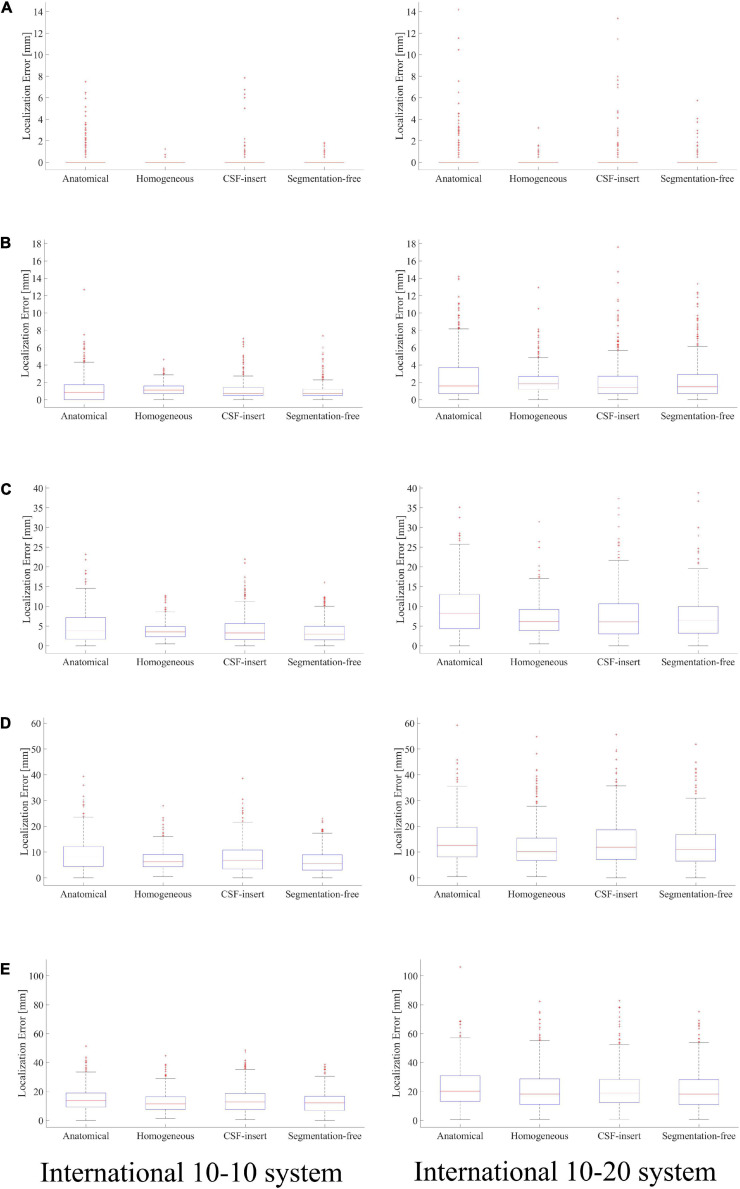
Localization error in different head models with SNR of, **(A)** ∞ dB, **(B)** 20 dB, **(C)** 10 dB, **(D)** 5 dB, and **(E)** 0 dB. Boxplots are calculated from 500 patterns of selected wave sources. In the inverse problem, measured potentials, and LFM are calculated in the same model.

Localization errors are shown in Figure 4 for the four head models and at five different noise levels. In the inverse problem, the measured potentials and LFM were calculated using the same model. Figure 4 demonstrates that the estimation error was less than 1 mm in all models for SNR = ∞. For the international 10–10 system, the mean localization error was 0.33, 0.06, 0.16, and 0.09 mm in the anatomical, homogeneous, CSF-insert, and segmentation-free models, respectively. The mean localization errors are presented in Table 4 and *t*-test results is shown in Table 5A.

**TABLE 4:** Mean localization error of results presented in Figure 4.

Model		SNR (dB)				
		∞	20	10	5	0
10–10 System	Anatomical	0.33 ± 0.94	1.18 ± 1.43	4.88 ± 3.99	8.95 ± 6.04	14.86 ± 8.51
	Homogeneous	0.06 ± 0.18	1.22 ± 0.65	3.88 ± 2.15	7.02 ± 3.81	12.80 ± 7.43
	CSF-insert	0.16 ± 0.70	1.03 ± 1.11	4.06 ± 3.41	7.67 ± 5.47	13.96 ± 8.78
	Segmentation-free	0.09 ± 0.28	0.96 ± 0.92	3.63 ± 2.78	6.54 ± 4.45	12.59 ± 7.51
10–20 System	Anatomical	0.35 ± 1.25	2.49 ± 2.67	9.13 ± 6.08	14.30 ± 8.84	23.37 ± 13.60
	Homogeneous	0.08 ± 0.27	2.13 ± 1.44	7.00 ± 4.16	12.11 ± 8.13	21.10 ± 13.69
	CSF-insert	0.25 ± 1.14	2.10 ± 2.30	7.58 ± 6.13	13.48 ± 8.82	21.75 ± 13.85
	Segmentation-free	0.14 ± 0.52	2.20 ± 2.32	7.30 ± 5.33	12.45 ± 8.22	20.87 ± 13.21

**TABLE 5:** *p*-Values obtained from pairwise two-sample two-tail *t*-test for localization error with all SNR values shown in **(A)** Figure 4 and **(B)** Figure 7.

	Anatomical	Homogeneous	CSF	Seg.-free
**(A)**				
Anatomical		0.000	0.005	0.000
Homogeneous	0.000		0.075	0.718
CSF	0.001	0.042		0.153
Seg.-free	0.000	0.169	0.001	
**(B)**				
Anatomical		0.000	0.000	0.000
Homogeneous	0.000		0.000	0.000
CSF	0.640	0.002		0.000
Seg.-free	0.000	0.000	0.000	

*Upper triangle is for the 10–20 system and lower triangle for 10–20 system.*

Comparing the results for the homogeneous and segmentation-free models, localization accuracy is almost the same for all noise conditions. In 10–10 system, all error means are significantly different except for the homogenous/segmentation-free models. On the other hand, in 10–20 system, no significant difference was observed between the homogenous and segmentation-free models and between CSF-insert and segmentation-free models. In the condition SNR = ∞, the averaged localization errors were comparable to each other for all models. In addition, the mean localization error is less significant for the international 10–10 system than for the international 10–20 system in all cases. The mean distance between the electrodes for the head models, which is similar for all the models, was 57.1 and 28.5 mm in the international 10–20 system and international 10–10 system, respectively.

In our simulations, the calculation time of the inverse problem was 14.2 s per source localization with an Intel Xeon E5 2643 v4 at 3.40 GHz running Windows Server 2019 at 3.40 GHz running CentOS 7.5 (6 cores, 12 threads, 512 GB RAM).

**FIGURE 5:**
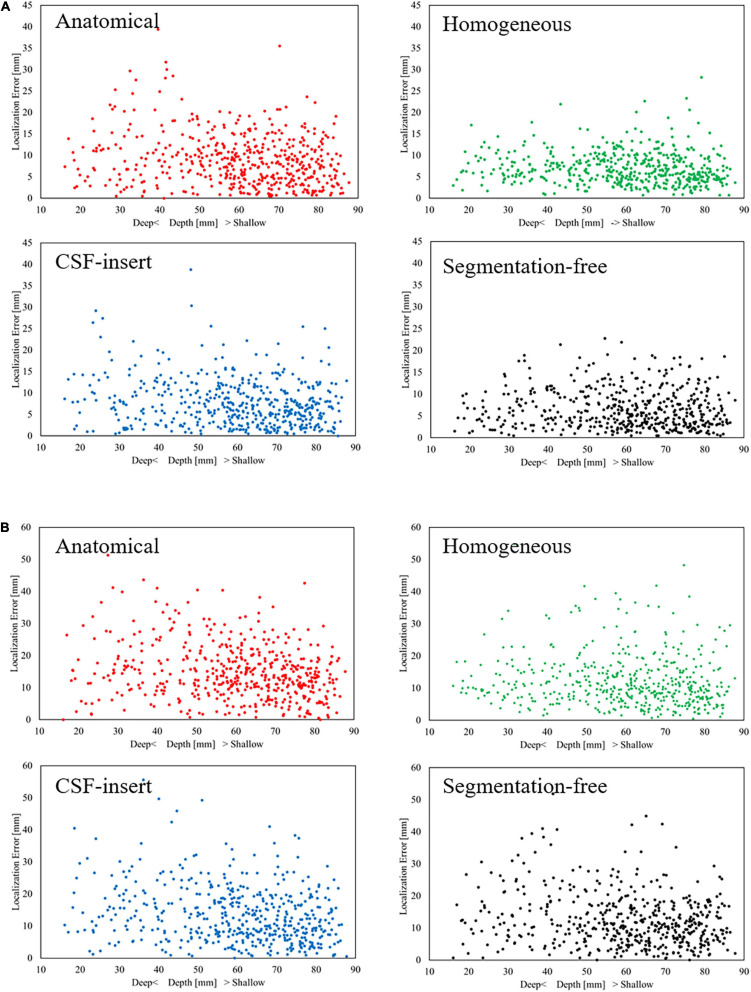
Relationship between depth and localization error with **(A)** International 10–10 system and **(B)** International 10–20 system in different head models for SNR = 5 dB. Each dot represents the localization error at 1 of the 500 selected test sources.

Figure 5 presents the relationship between the depth of the selected test source and the localization error in each head model for SNR = 5 dB with the international 10–10 and 10–20 systems, respectively. In this figure, a center of the brain is defined as the intersection of the lines under Cz, T7, and T8 in the international 10–10 system (Guadagnin et al., 2016). The distance between the center and the selected test source was defined as the depth. The smaller the depth value, the deeper the selected test source inside the brain. It can be observed that the localization error is insensitive to the source depth for all models and electrode patterns. For the international 10–10 system, the corresponding correlation coefficients between the depth and localization error were −0.1762, −0.0950, −0.1873, and −0.0558 in the anatomical, homogeneous, CSF-insert, and segmentation-free models, respectively, while their corresponding values were −0.2604, −0.1001, −0.2233, and −0.1895 for the 10–20 system.

**FIGURE 6:**
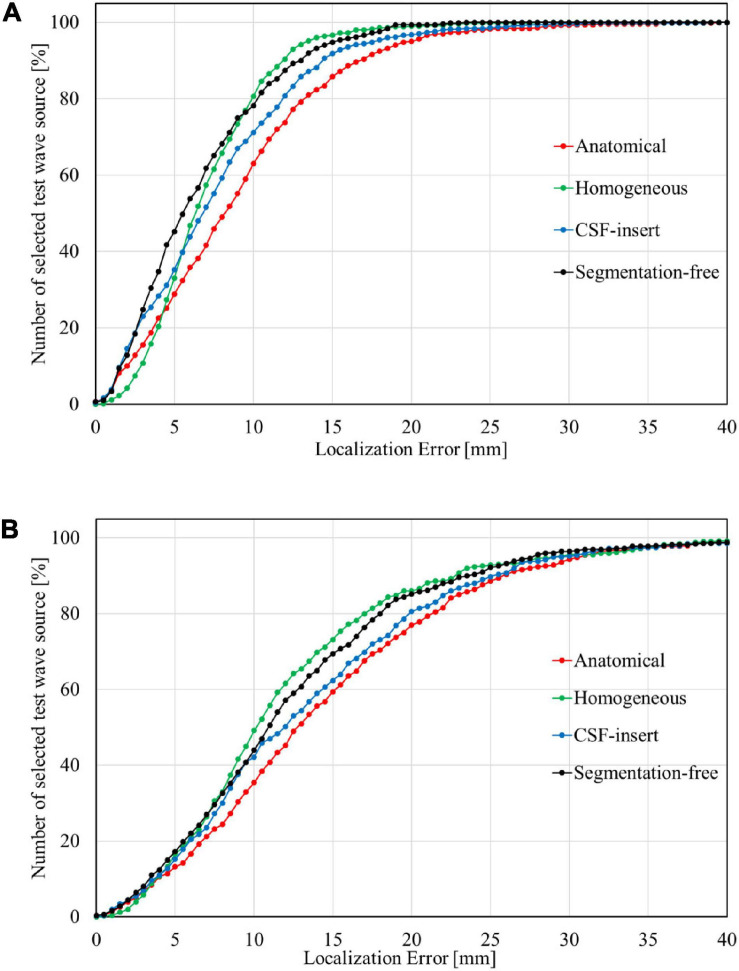
Cumulative distribution of localization error with **(A)** 10–10 system and **(B)** 10–20 system in different head models for SNR = 5 dB. The bin size was 0.5 mm.

Figure 6 presents the cumulative distribution of localization error in each head model for SNR = 5 dB with the international 10–10 and 10–20 systems. The localization error improved with an increase in the number of electrodes. The maximum localization errors in the anatomical, homogeneous, CSF-insert, and segmentation-free models were 39.40, 27.96, 38.60, and 23.00 mm, respectively, for the international 10–10 system, and 59.23, 54.78, 55.60, and 51.85 mm for the 10–20 system. In addition, the percentage of localization errors less than 10 mm was 63.0, 80.6, 71.2, and 78.2% for the international 10–10 system, and 35.4, 49.2, 42.2, and 44.0% for the 10–20 system.

**FIGURE 7:**
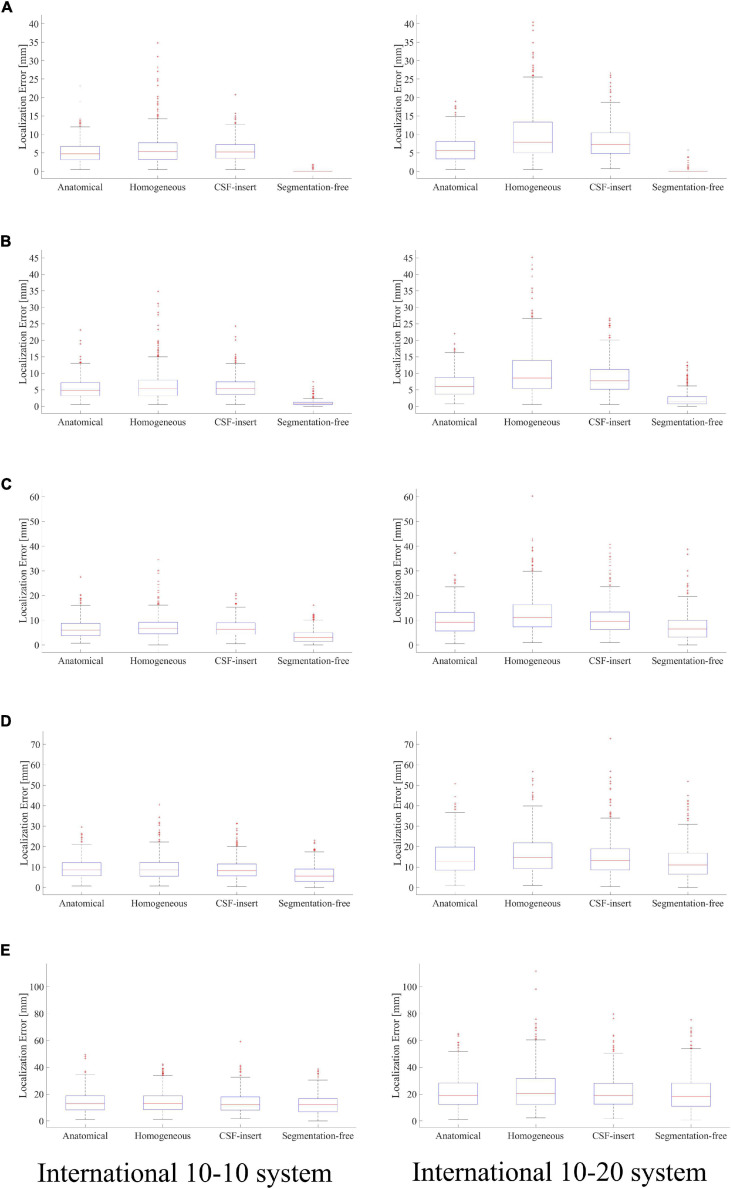
Localization error in different head models with SNR of, **(A)** ∞ dB, **(B)** 20 dB, **(C)** 10 dB, **(D)** 5 dB, and **(E)** 0 dB. Boxplots are calculated from 500 patterns of selected wave sources. In the inverse problem, measured potentials are fixed at the value calculated in the segmentation-free model. LFM is calculated in each model.

Localization errors are presented in Figure 7 for the four different models and five different noise levels. In the inverse problem, the measured potentials were fixed at the value calculated using the segmentation-free model. The LFM was calculated for each model. Figure 7 shows that when the result of the segmentation-free model is a reference value, the difference in the average localization error is the highest in a noise-free environment. For the international 10–10 system, the mean localization error was 5.22, 6.21, 5.67, and 0.09 mm in the anatomical, homogeneous, CSF-insert, and segmentation-free models, respectively. The mean localization errors are presented in Table 6. In all cases, the homogeneous model exhibited the largest error. This result was particularly apparent in the international 10–20 system. The results of the corresponding *t*-test are shown in Table 5B. As seen from this table, localization error means are significantly different for all models except for the anatomical and CSF-insert models in 10–10 system.

**TABLE 6:** Mean localization error of results presented in Figure 7.

Model		SNR (dB)				
		∞	20	10	5	0
10–10 System	Anatomical	5.22 ± 2.89	5.45 ± 3.07	6.67 ± 3.83	9.27 ± 5.12	14.13 ± 8.00
	Homogeneous	6.21 ± 4.49	6.28 ± 4.52	7.44 ± 4.61	9.49 ± 5.61	14.39 ± 8.08
	CSF-insert	5.67 ± 3.01	5.80 ± 3.22	6.88 ± 3.49	9.05 ± 4.95	13.72 ± 7.74
	Segmentation-free	0.09 ± 0.28	0.96 ± 0.92	3.63 ± 2.78	6.54 ± 4.45	12.59 ± 7.51
10–20 System	Anatomical	6.12 ± 3.44	6.54 ± 3.63	9.95 ± 5.56	14.55 ± 8.39	21.29 ± 11.94
	Homogeneous	9.96 ± 7.01	10.55 ± 7.33	12.80 ± 7.95	16.79 ± 10.02	23.68 ± 15.02
	CSF-insert	7.93 ± 4.36	8.57 ± 4.63	10.56 ± 6.22	14.91 ± 9.29	21.57 ± 12.19
	Segmentation-free	0.14 ± 0.52	2.20 ± 2.32	7.30 ± 5.33	12.45 ± 8.22	20.87 ± 13.21

### Extensibility of Our Method Using the Matching Pursuit Method

**FIGURE 8:**
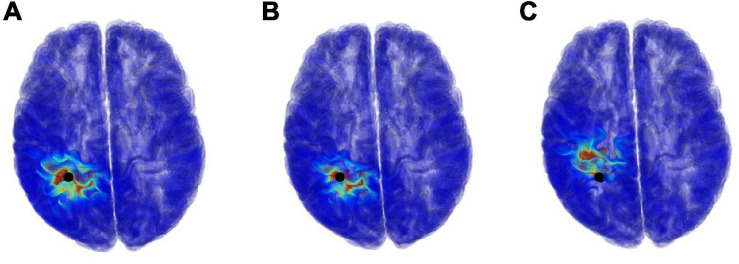
Examples of current density distribution results, which is used segmentation free model and varying the SNR, **(A)** ∞ dB, **(B)** 5 dB, and **(C)** 0 dB. The black points denote true position of electric dipole.

Our proposal can estimate not only the source location, but also the current density (electric field) distributions. The electric field distribution can be generated by calculating the position of electric dipole using MP with LFM and scalar potential φ, and then analyzing it using the SPFD method. Figure 8 shows examples of the current density distribution results when using a segmentation free model for different SNRs with respect to the potential φ. For SNR of 5 and 0 dB, the error distance between the true and estimate location was 4.77 and 12.75 mm, respectively.

### Comparison With Previous Studies

Table 7 summarizes the localization performance of the proposed method and that of the methods presented in previous studies. Previously, forward and inverse problems were solved for low-resolution models, as reported earlier (Grech et al., 2008). In recent years, high-resolution models have been considered while solving forward problems (Wang and Ren, 2013; Song et al., 2015). Wang and Ren calculated the same scenarios in the forward problem and in a solution space. The limitation of their proposal is that estimations can only be performed on the location for which the forward problem was solved. Furthermore, Song’s study adopted sLORETA as the inverse problem solver, which applied a relatively coarse (low-resolution) mesh. Consequently, the estimated error is considered to be non-existent if there is an estimated position in the low-resolution volume, which means that the error is potentially underestimated.

**TABLE 7:** Localization performance and specifications of related studies.

	Proposed		Song et al., 2015	Wang and Ren, 2013	Grech et al., 2008
Forward problem solver	FDM 0.5 mm × 0.5 mm × 0.5 mm		FDM 1 mm × 1 mm × 1 mm	BEM 1 mm × 1 mm × 1 mm	BEM 5 mm × 5 mm × 5 mm
Number of forward problem	74		70	8000	19,290
Localization method	Matching pursuit		sLORETA	Levenberg–Marquardt algorithm	sLORETA
Solution space	9635433 0.5 mm× 0.5 mm × 0.5 mm		7341 7 mm × 7 mm × 7 mm	8000 1 mm × 1 mm × 1 mm	19290 5 mm × 5 mm × 5 mm
Number of electrodes	75		71	31	32
Noise level (dB)	20	10	20	10	10
Error (mm)	0.96 ± 0.92	3.63 ± 2.78	0.53 ± 2.0	7.55	6.7 ± 1.0

*The number of forward problems is the number of computations needed for developing the lead field matrix.

A straightforward comparison of these methods is not feasible because, unlike previous studies, we solved the forward and inverse problems via high-resolution models. Although the mean error obtained herein is greater than that obtained (Song et al., 2015) by 0.43 mm, the standard deviation of the error was 1.08 mm lower.

## Discussion

In this study, we investigate EEG source localizations in different head models. Unlike in previous studies (Akalin Acar and Makeig, 2013; Montes-Restrepo et al., 2014), biological tissues were comprehensively examined using a 0.5-mm resolution in this study. This high-resolution is essential and necessary to consider the effect of CSF on localization accuracy. In addition, a large number (more than three million) of gray matter voxels were employed, much higher than those used in conventional methods (1000–50,000 voxels) (Grech et al., 2008; Wang and Ren, 2013; Song et al., 2015). Furthermore, a spatially dependent conductivity model created using machine learning was adopted to enable more realistic representation of head tissue conductivity. The noise immunity of these four models was investigated via Monte Carlo simulations, in which Gaussian white noise was introduced at five different noise levels. Also, we examined the estimation performance when the number of electrodes was varied.

In conventional head modeling for EEG studies, electrical conductivity was commonly set to be uniform within the same tissue. The standard process required a segmentation of different head tissues which is known as time consuming task. Recently, we developed a fast deep learning approach that can estimate non-uniform dielectric properties directly from MRI images (Rashed et al., 2020). Using this approach, a segmentation-free head model can be generated and used in this study. The results obtained are presented in Figure 3 and Table 3, and the characteristics of the segmentation-free model are most similar to those of the homogeneous model. This is because the segmentation-free model, unlike the anatomical and CSF-insert models, exhibits a smoother conductivity distribution (except around CSF in the CSF-insert model). In addition, the correlation coefficient of the potentials on the electrodes with the anatomical model was improved from 0.850 to 0.896 by considering the CSF in the homogeneous model. This result indicated that the accurate modeling of the CSF may contribute to the localization accuracy, as suggested based on forward problems in previous reports (Ramon et al., 2004; Azizollahi et al., 2016; Stenroos and Nummenmaa, 2016). Because the CSF exhibits the highest conductivity value among the head model tissues, the current in the gray matter is diffused, and its direction is guided by the CSF. Furthermore, the CSF is a thin layer; hence, a high spatial resolution (0.5 mm in this study) was required for reliable modeling. Even considering one cell (0.5 mm), the current may concentrate around there due to high conductivity. The CSF distribution can be in region with thickness thinner than 1 mm; however, its thickness depends on age (Haeussinger et al., 2011).

In an earlier study, the effect of human head simplification on EEG source localization was investigated (Neugebauer et al., 2017). The results demonstrated the importance of using detailed modeling of brain structures such as CSF, white matter and gray matter. In our study, we demonstrated the variability in electrical conductivity of different tissues on localization accuracy using different models. In particular, the error caused by abrupt change of conductivity values around region boundaries was identified.

This uncertainty caused by the discontinuity can also be explained from the viewpoint of physics. As the same physical law is used in several applications such as EEG, one can clearly expect that the definition of tissue boundaries (at which an abrupt change of electrical conductivity occurs) is a dominant error source (computational artifact). This is significant especially around the complicated tissue distributions, resulting in unsmoothed current density distribution (Stuchly and Dawson, 2000; Gomez-Tames et al., 2018; Soldati and Laakso, 2020). It is demonstrated that increasing model resolution (more fine details) can lead to improve the accuracy of the electric field computations. However, finite difference computation used a computational residual for truncating the computation, and thus local error may not be neglected (Laakso and Hirata, 2012a). In addition, we evaluated the influence of segmentation error on the distribution of electric field in the brain for non-invasive brain stimualtion (Rashed et al., 2021). Although it is not EEG study, but it clearly demonstrates that small change in segmentation (few voxels) of head components (such as CSF) leaded to a remarkable change in the local electric field distribution.

Comparing the results of the anatomical model and segmentation-free model for SNR = 0 dB, the averaged localization errors are 14.86 and 12.59 mm in the international 10–10 system. This means that error in source localization is potentially attributed to an abrupt change in conductivity derived from the tissue boundary. As presented in Table 4, the proposed method achieved the same level of accuracy as that of previous studies (Song et al., 2015; Sohrabpour et al., 2016). These results highlight the importance of using segmentation-free models. In one hand, it can be quickly generated from anatomical images. On the other hand, it reduces localization error caused by the sudden change of tissue electrical conductivity around boundaries. To the best of author’s knowledge, this approach is original contribution of this study and has not been discussed in the EEG context before.

The correlation coefficient between the depth and localization error was insignificant. In previous studies (Wang et al., 1992; Dale and Sereno, 1993; Matsuura and Okabe, 1995), it was determined that the MN method makes it difficult to estimate the depth of the brain. Therefore, various methods have been developed to address this challenge. However, these methods accrue high computational costs compared to the MN method. In contrast, the proposed method provides the same level of estimation accuracy for all brain regions using a simple algorithm. We confirmed that the localization error was significant when the interval between the electrodes was doubled (Figure 6). The percentage that exhibited an error less than 10 mm, which may approximately correspond to the width of the gyrus (Freeman and Quiroga, 2012), differed by 30% between the international 10–10 and 10–20 systems at SNR = 5 dB. In this study, white Gaussian noise and model conductivity were the only factors that triggered localization errors. In addition, there are several factors influencing localization estimation using experimental results (e.g., electrode position and head modeling). Therefore, it may be impractical to apply clinical scenarios, where additional error sources are case-specific.

From Figure 7 and Table 6, comparing the results of the anatomical, CSF-insert, and homogeneous models, the averaged localization and maximum errors of the homogeneous model are the worst in almost all cases. In addition, the anatomical and CSF-insert exhibit similar results. In particular, the results of the noise-free condition represent an error that occurs only in the conductivity distribution; this is because all conditions are the same, except for the LFM. This result also indicates that the consideration of the CSF is essential for representing a realistic human head, as illustrated in Figure 3. However, for low SNRs, the differences in conductivity become increasingly obscure, thus suggesting that the importance of head modeling varies significantly based on the experimental environment.

Comparing the estimated current distribution with SNR of ∞ and 5 dB, the estimated regions were close to each other (5 mm). On the other hand, comparing the results with SNR of ∞ and 0 dB, the difference became larger than 10 mm. Though only a distribution of single source was presented here for simplicity, our method can generally estimate multiple sources in iterative manner; first, a single dipole location is estimated. Therefore, with the residual computed using Eq. (4) for the first source estimation, the second source location can be estimated using matching pursuit method in the same manner. Even for multiple sources, the field distribution can be generated with the SPFD method. Thus, the proposed method can estimate the current density distribution from any observed potentials.

## Conclusion

In this study, we proposed an EEG source localization algorithm based on the adoption of fast high-resolution computational analysis (0.5 mm) for a forward problem and sparse modeling for an inverse problem. This modeling setting was employed to consider the CSF layer, which has been suggested to be important in previous studies (Ramon et al., 2004; Azizollahi et al., 2016; Stenroos and Nummenmaa, 2016). This enabled us to perform source localization using a high-resolution model with a reasonable computational cost. In addition, considering the different approaches employed in the development of head models, we determined that an abrupt change in conductivity at the tissue boundary is the primary factor that triggers localization errors in high-SNR conditions. Head model with smooth conductivity, which are developed from medical images directly with machine learning suppressed the error caused by the discontinuity of the conductivity. The proposed algorithm with new head modeling achieved an estimation error of less than 10 mm for SNR = ∞ in the international 10–10 system. For a high SNR, the average localization error was comparable to those in previous studies.

## Data Availability Statement

The datasets presented in this article are not readily available. The ethical approval for using MRI data has been obtained at Nagoya Institute of Technology (2019-014). Based on the permission, these images are not available for other research purposes. Requests to access the datasets should be directed to AH, ahirata@nitech.ac.jp.

## Author Contributions

AH conceived and designed the study. TM and ER developed the numerical algorithm. TM and SM conducted the numerical experiments. TM and ER processed the data. All authors analyzed the data, wrote the manuscript, and read and approved the manuscript.

## Conflict of Interest

The authors declare that the research was conducted in the absence of any commercial or financial relationships that could be construed as a potential conflict of interest.
